# Sleep Apnea and Inflammation – Getting a Good Night’s Sleep with Omega-3 Supplementation

**DOI:** 10.3389/fneur.2013.00193

**Published:** 2013-12-03

**Authors:** Fulvio A. Scorza, Esper A. Cavalheiro, Carla A. Scorza, José C. F. Galduróz, Sergio Tufik, Monica L. Andersen

**Affiliations:** ^1^Disciplina de Neurologia Experimental, Universidade Federal de São Paulo/Escola Paulista de Medicina (UNIFESP/EPM), São Paulo, Brazil; ^2^Departamento de Psicobiologia, Universidade Federal de São Paulo/Escola Paulista de Medicina (UNIFESP/EPM), São Paulo, Brazil

**Keywords:** obstructive sleep apnea, omega-3, cardiovascular, inflammation, TNF-α

## Abstract

Obstructive sleep apnea (OSA) is a multifactorial sleep disorder associated with an increased risk of cardiovascular mortality and morbidity. Several mechanisms have been proposed to explain the association between OSA and cardiovascular dysfunction. One of the proposed mechanisms is an inflammatory response to OSA mediated by tumor necrosis factor (TNF-α). Patients with OSA have higher plasma, serum, and intracellular levels of TNF-α, which may be reduced after apnea treatment with continuous positive airway pressure (CPAP). Because TNF-α plays an important role in OSA related cardiovascular morbidity, the present review aims to identify other preventive measures, in addition to CPAP, that may minimize the inflammatory process in OSA and consequently the risk of premature death due to cardiovascular dysfunction. Thus, we hypothesized that a nutritional immunology profile, i.e., supplementation with omega-3 fatty acids, may be valuable for individuals with OSA.

Obstructive sleep apnea (OSA), a common form of sleep-disordered breathing, is a multifactorial sleep disorder associated with several risk factors, including excess body weight, male gender, older age, neck circumference, body mass index, and high blood pressure (1, 2). OSA affects up to 32.8% of the population (3) and, as demonstrated in the Sleep Heart Health study, is linked to an increased risk of cardiovascular mortality and morbidity (4). Population-based longitudinal studies have shown that individuals with severe OSA have a threefold greater risk of all-cause mortality and a higher cardiovascular mortality at 18-year follow-up (5–7). In addition, OSA has been independently associated with specific cardiovascular outcomes such as hypertension (8), stroke (9, 10), myocardial ischemia (11, 12), and arrhythmias with an increased risk for sudden cardiac death (13, 14). Several mechanisms have been proposed to explain the association between OSA and cardiovascular dysfunction (15). Interestingly, the accumulated evidence based on clinical studies as well as animal models and cell culture indicates that inflammatory cytokines, especially tumor necrosis factor (TNF, also known as TNF-α), play an important role in OSA related cardiovascular morbidity (15). In 1975, TNF-α was identified as an endotoxin induced glycoprotein that caused hemorrhagic necrosis of sarcomas that had been transplanted into mice. Human TNF was cloned 10 years later (16–18). Briefly, TNF-α is an inflammatory cytokine produced by macrophages/monocytes during acute inflammation and is responsible for a diverse range of signaling events within cells that lead to necrosis or apoptosis (19). In addition to its involvement in a diverse range of inflammatory, infectious, and malignant conditions, the importance of TNF-α has been highlighted by the efficacy of anti-TNF antibodies or soluble TNF receptors (TNFRs) in controlling inflammatory conditions (18). Several important discoveries have been made in elucidating the precise role of TNF-α in the cardiovascular pathogenesis of OSA. Entzian et al. (20) demonstrated that the circadian rhythm of TNF-α release was significantly disturbed in OSA patients, suggesting that TNF-α could play a pathophysiologic role in OSA. After this discovery, TNF-α levels were extensively studied in individuals with OSA (15). In most of these studies, TNF-α was measured in plasma or serum; however, in several studies cell-specific levels were also evaluated (15, 21, 22). In 2003, Dyugovskaya et al. (21) characterized the cytokine profile of gammadelta T cells in patients with OSA and control subjects. The major finding regarding OSA gammadelta T cells was a significant increase in the intracellular content of proinflammatory cytokines TNF-α in OSA individuals when compared with the control group. A year later, it was shown that individuals with moderate-to-severe OSA had spontaneous production of TNF-α by monocytes and elevated serum levels of TNF-α (23). Ryan et al. demonstrated an important association between TNF-α and OSA severity, as serum levels of TNF-α was higher in subjects with OSA than in subjects without OSA (24). Moreover, other authors have also demonstrated that continuous positive airway pressure (CPAP) therapy decreased the TNF-α level in OSA patients (25). Following this line of reasoning, Dorkova et al. (26) determined the effects of 8 weeks of CPAP therapy on inflammation in patients with severe OSA. They provide additional evidence that OSA caused an increase in TNF-α and also concluded that compliance with a CPAP regimen can improve the expression of this inflammatory cytokine. Furthermore, evaluating obese Asian Indians with OSA, Bhushan et al. (27) clearly demonstrated that the frequency of the TNF-α (−308A) allele and the serum TNF-α level was significantly higher in OSA (27). Despite this evidence the role of TNF in OSA there is still controversy in the scientific literature on this aspect. One possible explanation pair these divergences could be related to disease duration, intensity, patient age, among other factors.

Based on these findings and the fact that TNF-α plays an important role in OSA related cardiovascular morbidity, we aimed to identify other preventive measures, aside from CPAP (successful treatment with CPAP appears to at least partially abrogate this risk) (28), that can minimize the inflammatory process in patients with OSA and thereby prevent possible cardiovascular dysfunction that may increase the risk of premature death. A possible alternative therapy is nutritional immunology, i.e., supplementation with omega-3 fatty acids (omega-3 FAs). For this proposal, several lines of evidence should be explored. First, the beneficial effects of omega-3 FAs in the cardiovascular system have been described since the late 1970s. It was demonstrated that the low prevalence of cardiac diseases in Eskimos might be due to their high dietary intake of omega-3 FAs (29). Currently, several studies have clearly demonstrated that omega-3 FAs can help prevent coronary heart disease, reduce arrhythmias and thrombosis, lower plasma triglyceride levels, and reduce blood clotting tendency (30). However, there is still controversy regarding the real cardiovascular benefits of omega-3 FAs. According to Barrett (31), the majority of epidemiological studies and interventions demonstrate favorable cardiovascular outcomes with omega-3 FAs; however, Galli and Brenna (32) have recently criticized the methodological meta-analysis and concluded that omega-3 FAs were not effective in preventing cardiac disorders.

Second, the relationship between omega-3 FAs and OSA is still rarely evaluated and described in the literature. One of the most relevant studies on this topic was conducted by Ladesich et al. (33). In this study, the authors investigated the relationship between omega-3 fatty acid docosahexaenoic acid (DHA) levels in red blood cells (RBC) and OSA severity in 350 sequential patients undergoing sleep studies. The authors defined the severity categories as none/mild, moderate, and severe based on apnea hypopnea index scores of 0–14, 15–34, and >34, respectively (33). Briefly, they demonstrated that RBC DHA was inversely related to OSA severity and that for each 1-SD increase in DHA levels, a patient was approximately 50% less likely to be classified with severe OSA (33). Thus, the authors concluded that disordered membrane fatty acid patterns may play a causal role in OSA and that the assessment of RBC DHA levels might help in the diagnosis of OSA (33). We fully agree with the authors’ conclusions, and our research group recently postulated that scientists and clinicians should work together in a worldwide network focused on basic scientific research programs and clinical studies to accurately establish the use of omega-3 FAs for at risk individuals to prevent sudden death in OSA patients (34).

Third, it is well documented that chronic inflammation is a characteristic of severe chronic heart failure (CHF), and inflammatory cytokines have been shown to reduce left ventricular (LV) function, promote LV remodeling, and deteriorate endothelial function (35–37). Thus, as recent published studies have accurately demonstrated, supplementation with omega-3 FAs improves systolic LV function and endothelial function as well as decreases markers of inflammation in CHF of non-ischemic origin (37) and other clinical conditions (38, 39). Therefore, it is entirely reasonable to postulate that omega-3 FA supplementation will be useful in minimizing the inflammatory cascade and therefore improving cardiac function in individuals with OSA.

In addition to the above points, omega-3 FAs are well tolerated with minimal adverse effects (40). As noted, the human body cannot synthesize omega-3 FAs; therefore, it must be ingested from the diet. According to the vast literature, seafood (commonly found in fish and fish oil) is the only food that provides large amounts of omega-3 FAs (30, 41). The best seafood choices are wild salmon, anchovies, sardines, trout, herring, and tuna (30, 41). Due to the importance of the consumption of fish rich in omega-3 FAs, international guidelines have suggested that the general population should consume at least 250 mg/day of long-chain omega-3 FAs or at least two servings/week of oily fish (42). For individuals who want a contaminant-free diet but would like to enjoy the benefits of omega-3 FAs, fish oil supplements, or foods such as walnuts or oils (flax, canola, and soybean) can be consumed (43, 44).

Overall, it is clear that inflammation (especially increased levels of TNF-α) plays an important role in OSA related cardiovascular morbidity (45). Due to the beneficial actions of omega-3 FAs for the cardiovascular system and in minimizing the inflammatory process, our research group has hypothesized that omega-3 FA supplementation may be able to reduce cardiac dysfunction and even the occurrence of premature death in patients with OSA.

We believe that new experimental, epidemiological, and clinical studies should be evaluated to establish the relationship between OSA, inflammation, and omega-3 FAs. In the meantime, we should be optimistic and remember these words by the American writer and philosopher Robert M. Pirsig: *For every fact there is infinity of hypotheses*.

## Author Contributions

All authors were involved in writing the paper and had final approval of the submitted and published versions.

## Conflict of Interest Statement

The authors declare that the research was conducted in the absence of any commercial or financial relationships that could be construed as a potential conflict of interest.
